# Electrical impulse effects on degenerative human annulus fibrosus model to reduce disc pain using micro-electrical impulse-on-a-chip

**DOI:** 10.1038/s41598-019-42320-9

**Published:** 2019-04-09

**Authors:** JaeHee Shin, MinHo Hwang, SeungMin Back, HyoGeun Nam, ChangMin Yoo, JeongHun Park, HyeongGuk Son, JaeWon Lee, HyunJung Lim, KwangHo Lee, HongJoo Moon, JooHan Kim, HanSang Cho, Hyuk Choi

**Affiliations:** 1 Department of Medical Sciences, Graduate School of Medicine, Korea University, Seoul, Korea; 2 Department of Advanced Material Science and Engineering, College of Engineering, Kangwon National University, Chuncheon, 25561 Korea; 3 Department of Neurosurgery, Guro Hospital, College of Medicine, Korea University, Seoul, Korea; 4 Department of Mechanical Engineering and Engineering Science, Department of Biological Sciences, Center for Biomedical Engineering and Science, Nanoscale Science Program, University of North Carolina at Charlotte, North Carolina, 28223 USA

## Abstract

Electrical stimulation of cells and tissues for therapeutic benefit is a well-established method. Although animal studies can emulate the complexity of an organism’s physiology, lab-on-a-chip platforms provide a suitable primary model for follow-up animal studies. Thus, inexpensive and easy-to-use platforms for *in vitro* human cell studies are required. In the present study, we designed a micro-electrical impulse (micro-EI)-on-a-chip (micro-EI-chip), which can precisely control electron density and adjust the frequency based on a micro-EI. The micro-EI-chip can stimulate cells at various micro-EI densities (0–500 mV/mm) and frequencies (0–300 Hz), which enables multiple co-culture of different cell types with or without electrical stimulation. As a proof-of-concept study, a model involving degenerative inflamed human annulus fibrosus (hAF) cells was established *in vitro* and the effects of micro-EI on inflamed hAF cells were evaluated using the micro-EI-chip. Stimulation of the cells (150 mV/mm at 200 Hz) inhibited the secretion of inflammatory cytokines and downregulated the activities of extracellular matrix-modifying enzymes and matrix metalloproteinase-1. These results show that micro-EI stimulation could affect degenerative diseases based on inflammation, implicating the micro-EI-chip as being useful for basic research of electroceuticals.

## Introduction

Symptomatic disc degeneration is a major cause of lower back pain^1^. In general, disc degeneration in the lumbar spine appears to result from inflammatory reactions involving various macrophages in the outer annulus fibrosus (AF)^2^. Human intervertebral discs (IVDs) are composed of AF cells and collagenous tissues that act as a cushion in the disc, absorbing dynamic biomechanical loads^3,4^. Generally, continuous biomechanical loading on AF tissue leads to damage and induces inflammatory reactions^5–7^. The latter subsequently induce ingrowth of nerves positioned near the outer AF tissue toward the inner AF tissue^8^. This mechanism contributes to disc degeneration and is accompanied by severe lower back pain^8,9^. Various studies have suggested that the major contributor to discogenic pain is neural pressure caused by herniation of nucleus pulposus tissue^10^. However, recent studies suggested that cytokines released from inflammatory reactions adjacent to the nerve roots affect the roots and lead to severe pain^11,12^. Damage to AF tissue is closely related to the early stage of inflammation; when AF cells are damaged, macrophages release pro-inflammatory cytokines, such as interleukin (IL)-1β and tumour necrosis factor-alpha (TNF-α), and induce inflammation^13–15^.

A logical approach to ameliorate or prevent symptomatic disc degeneration would be to stimulate the production of extracellular matrix (ECM) and/or to inhibit the activity of inflammatory mediators and matrix metalloproteinases (MMPs)^16,17^.

Electrical stimulation (ES) has been reported to affect cell migration, wound healing, and inflammation^18–20^. An *in vitro* study demonstrated that ES is involved in osteoblast differentiation of human mesenchymal stromal cells through vascular endothelial growth factor and bone morphogenetic protein-2 induction via the activation of mitogen-activated protein kinase and calcium channels^21^. Another study showed that ES significantly diminishes the gene expression of IL-17A, MMP-2, and nuclear factor-kappa B (NF-κB) induced by IL-1α in IVD cells^22^. An additional study observed ES-induced upregulation of disc-matrix macromolecular components, including sulphated glycosaminoglycan, aggrecan, and collagen type 2^23^. ES was also shown to modulate the expression of multiple wound healing genes, including tissue inhibitor of metalloproteinase (TIMP) and MMP, in human dermal fibroblasts^24^. Additionally, ES influences the polarization state of the cell membrane, causing changes in the cellular microenvironment^25^. The human body generates bioelectricity through ion channels, which perform various biological processes, in the range of 100–300 Hz^26–28^. ES has been applied to spinal cord injuries in an attempt to develop clinical therapies^29^. However, the precise mechanisms by which the response to this stimulation occurs remain poorly understood. Thus, we examined the effects of ES on pain-related factors in IVD cells^18^.

A previous study reported the inhibitory effect of ES toward inflammatory reactions induced by treatment with dilute recombinant IL-1β reagent^18^. However, this approach has limitations in mimicking the actual inflammatory mechanism of the human body because typical processes can produce inflammation based on complex interactions between damaged tissue and immune cells^2^. Additionally, the model described in the previous study had some limitations in explaining the signalling pathway related to major inflammatory mediators and persistence of ES effects. Accordingly, we designed and fabricated a precise device platform, termed a micro-electrical impulse (micro-EI)-on-a-chip (micro-EI-chip) that can ensure precise electron density and adjustable frequency based on the biphasic micro-EI. It also enables live multiple co-culture of different cell types with or without ES. Besides, we could take microscopic examination and run multiple assays, such as enzyme-linked immunosorbent assay (ELISA), lactate dehydrogenase (LDH) assay, and immunofluorescence quantification in a single batch as a single chipset contains 10 identical cell chambers.

In the present study, we used a micro-EI-chip system to investigate the effects of electrical impulses using a degenerative human AF model to reduce disc pain-related factors.

## Materials and Methods

### Deliberate micro-EI strength and frequency adjustment during micro-EI stimulation

The micro-EI-chip platform fabricated for micro-EI of the human AF (hAF) cells is comprised of a live cell imaging system and micro-EI-chip (Supplementary Fig. S1). The advantages of the micro-EI-chip are the adjustable micro-EI strength and frequency levels. The output voltage is fixed. However, because of the potentiometer, the resistance is adjustable and can regulate the micro-EI strength. The output micro-EI strength in the micro-EI-chip is in the range of 0–150 mV/mm. Furthermore, the output timing of the voltage can be adjusted using the Atmega128 (Mouser Electronics, Inc., Kwun Tong, KL, Hong Kong, China). Thus, frequency and duration are adjustable in the micro-EI-chip. The Atmega128 is controlled by IAR Embedded Workbench software (IAR Systems, Uppsala, Sweden) and can be controlled in the range of 0–16 MHz. The voltage output was calibrated using a model DSO6014A oscilloscope (Agilent Technologies, Santa Clara, CA, USA) and a model 6485 picoammeter (Keithly Instruments, Inc., Cleveland, OH, USA) (Supplementary Fig. S2). During the cell culture process, the cellular environment was automatically controlled and maintained at 36.5 ± 0.5 °C, 70% humidity, and 5% CO_2_ concentration in air using an automatic control system (temperature controller and gas mixer). The pH was determined to be 7.35 using a pH meter (Mettler Toledo, Columbus, OH, USA).

### Availability of various molecular biological analyses in the micro-EI-chip

The frame of the micro-EI-chip was fabricated using a three-dimensional (3D) printer (3D Systems, Rockhill, SC, USA), in which medical grade material (VisiJet M3 Crystal, 3D Systems) was used for long term cell culture. The chip comprised a micro-electrical impulse generator at the centre, with the 10 cell culture systems located surrounding the centre. Each cell culture system had two separate chambers, with a hydrogel channel between them (Supplementary Fig. S3). In the cell culture system, one chamber was set up for macrophage cells and the other was cultured with AF cells, enabling observation of the interactions between macrophages and AF cells. This system allowed us to mimic the symptomatic disc degeneration at the micro scale. Thus, 10 samples of two different cell types can be observed and analysed in parallel.

Additionally, the micro-EI-chip was designed for micro-EI stimulation of various cell types and is easy to use because of the application of universally available materials, such as Indium Tin Oxide (ITO; Nippon Sheet Glass Co., Tokyo, Japan) glass and polydimethylsiloxane (PDMS)-coated gold/titanium (Au/Ti) patterned electrodes. The chip is optically clear, enabling microscopic examination. The Au-patterned electrodes were fabricated on the ITO glass via a photolithography process using an AZ-5214E photoresist (MicroChemicals GmbH, Ulm, Germany) with a lift-off process. The patterning was followed by deposition of a bilayer of 250/500 Å Ti/Au using an e-beam evaporation system, which was then coated with PDMS substrate.

### Isolation and culture of hAF cells

hAF cells were removed from the disc tissues of six male patients with degenerative spinal disease (Pfirrmann degenerative grades II–III) during elective surgery. This study was approved by the Korea University Hospital Institutional Review Board (KUGH170208-001), and informed consent was obtained from the subjects. All methods were performed in accordance with the guidelines and regulation of the Human Ethics Committee of the Korea University Hospital. Tissue samples were placed in Ham’s F-12 medium (Gibco-BRL, Grand Island, NY, USA) containing 5% foetal bovine serum (FBS; Gibco-BRL) and 1% penicillin/streptomycin (P/S; Gibco-BRL), and then washed with phosphate-buffered saline (Gibco-BRL). AF regions were minced and digested for 1 h at 37 °C with gentle agitation in F-12 medium supplemented with 5% FBS, 1% P/S, and 0.2% pronase (Calbiochem, La Jolla, CA, USA). The cells were then incubated overnight with 0.025% collagenase I (Roche Diagnostics, Mannheim, Germany). Subsequently, the cells were filtered through a sterile nylon mesh (70-µm pore size) and centrifuged (676 × g, 5 min). The pellet was resuspended in F-12 medium supplemented with 10% FBS and 1% P/S (culture medium) and cultured at 37 °C in a humidified atmosphere containing 5% CO_2_.

### Macrophage preparation and induction of inflammation in naïve hAF cells

Human monocytic THP-1 cells (ATCC TIB202; ATCC, Manassas, VA, USA) were seeded (1.0 × 10^4^ per channel) and cultured in the macrophage channel of the micro-EI-chip system at 37 °C in a CO_2_ incubator. THP-1 cells were cultured in Roswell Park Memorial Institute 1640 medium (ATCC) containing 10% FBS, 1% P/S, and 0.05 mM 2-mercaptoethanol (Sigma Chemical Co., St. Louis, MO, USA). Subsequently, these cells were stimulated with 160 nM phorbol myristate acetate (PMA) for 48 h to convert them into activated macrophage-like THP-1 cells, which can adhere to the substrate and secrete pro-inflammatory cytokines via NF-κB pathway activation^30–32^. After setting up the macrophage channel, naïve hAF cells were seeded (5.0 × 10^3^ per channel) and cultured in the IVD cells channel. hAF cells were cultured in F-12/Dulbecco’s modified Eagle medium containing 1% FBS and 1% P/S (low-serum medium). To create inflamed hAF cells, naïve hAF cells were exposed to pro-inflammatory cytokines from the macrophage channel. The soluble pro-inflammatory cytokines from the macrophage channel diffused into the hAF cells in the IVD cells channel and converted naïve hAF cells into inflamed hAF cells.

### Micro-EI stimulation

**Figure 1:**
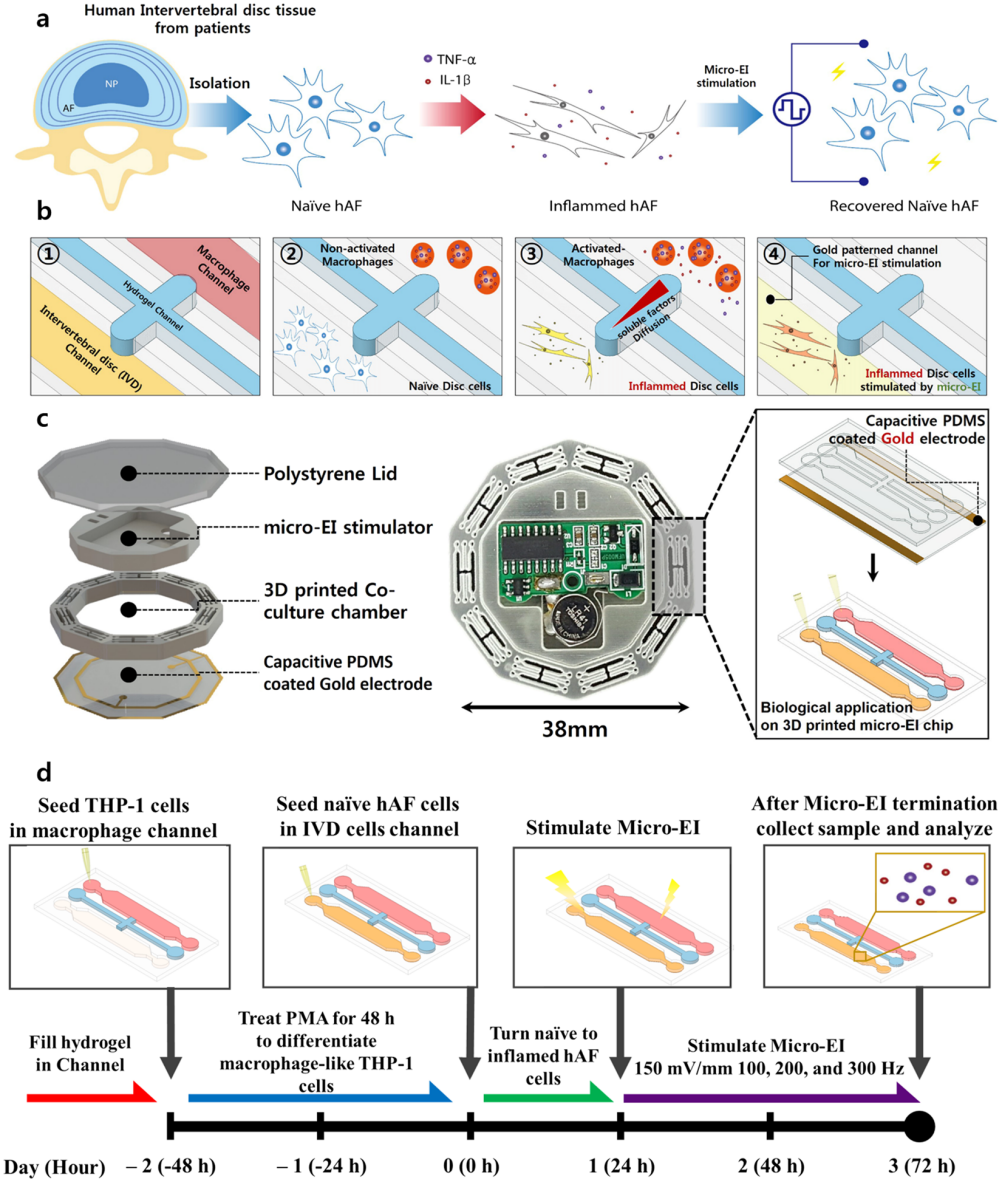
Summary of establishment of the degenerative inflamed hAF model by treatment with pro-inflammatory soluble factors and assessment of the effects of micro-EI on inflamed hAF in a micro-EI-chip. (**a**) Scheme of the experimental design. (**b**) (1) 3D hydrogel formation between the intervertebral disc cell and macrophage channel. (2) Human THP-1 cells (non-activated macrophages) treated with PMA to stimulate differentiation of activated-macrophages in the upper channel. After 48 h, the disc cells were seeded and cultured in the IVD cells channel. (3) Disc cells were stimulated by soluble factors derived from activated macrophages, which can induce inflammatory responses on disc cells. (4) Micro-EI was applied to the inflamed disc cells for 48 h. (**c**) Micro-EI-chip platform designed for culturing and stimulating hAF cells. (**d**) Whole experimental process. Abbreviations are; Micro-EI, micro-electrical impulse; hAFs, human annulus fibrosus cells; and PMA, phorbol myristate acetate.

While pro-inflammatory cytokines were fully diffusing for 24 h, inflamed hAF cells received either no stimulation or micro-EI stimulation (150 mV/mm at 100, 200, or 300 Hz) for 48 h (Fig. 1).

### Immunofluorescence (IF) staining

hAF cells were fixed with 4% paraformaldehyde and then permeabilized with 0.2% Triton X-100 in phosphate-buffered saline for 10 min, blocked with 3% bovine serum albumin for 1 h at 25 °C (room temperature), and incubated overnight at 4 °C with an anti-p50 antibody (Santa Cruz Biotechnology, Dallas, TX, USA). The hAF cells were then incubated with Alexa 555-conjugated anti-rabbit IgG secondary antibody (Santa Cruz Biotechnology) and Alexa 488-conjugated phalloidin (Invitrogen, Carlsbad, CA, USA). Finally, cells under coverslips were counterstained with 4′,6-diamidino-2-phenylindole (Santa Cruz Biotechnology). The stained cells were imaged and examined using an LSM 700 confocal laser scanning microscope (Zeiss, Oberkochen, Germany).

### ELISA

The concentration of inflammatory mediators in the culture supernatant (conditioned medium; CM), including IL-6, IL-8, and ECM-modifying enzymes, including MMP-1 and TIMP-2, were quantified using commercially available ELISA kits (R&D Systems, Minneapolis, MN, USA).

### MMP collagen zymography assay

A collagen zymography assay was used to detect MMP-1 activity. The culture supernatant was centrifuged at 9360 ×*g* for 1 min at 4 °C to dispose of cellular debris. The protein concentration was determined using a NanoDrop spectrophotometer (Thermo Fisher Scientific, Waltham, MA, USA). The samples were mixed with an equal volume of 2× zymogram sample buffer (Komabiotech, Seoul, Korea) and 20 µL of the mixture was loaded onto 0.4 mg/mL collagen**–**10% polyacrylamide gels. After electrophoresis, the gels were washed twice with 2.5% Triton X-100 for 30 min with shaking to remove the sodium dodecyl sulphate and renature the MMP-1 protein in the gels. Renatured gels were incubated with developing buffer consisting of 100 mM Tris-HCl (pH 8.0), 5 mM CaCl_2_, 0.005% Brij-35, and 0.001% NaN_3_ overnight at 37 °C. Gels were stained with 0.25% Coomassie brilliant blue G-250 (in 50% methanol with 10% acetic acid). Proteinase activity was determined to detect unstained regions. Finally, the gels were dried for 2 h in a gel dryer (Bio-Rad Laboratories, Hercules, CA, USA).

### LDH assay to detect cytotoxicity

To evaluate the possible toxic effects of micro-EI stimulation on cells, CM was collected after micro-EI stimulation for 48 h. LDH was then assayed using an LDH assay kit (Roche).

### Analysis of cell kinetics

Automated microscopic time-lapse images of individual hAF cells grown for 72 h were acquired using a live cell imaging system (Nowon, Seoul, Korea). We also measured the cell cytoplasmic area and migration speed in the acquired cell images using ImageJ software (NIH, Bethesda, MD, USA).

### Finite Element Analysis

We determined the diffusion rate pattern from the macrophage channel to the IVD cell channel in the micro-EI chip device platform using finite element analysis with Multiphysics software (COMSOL, Burlington, MA, USA). The boundary condition for the pro-inflammatory cytokines diffusion model included ambient temperature and atmospheric pressure. Transport properties applied Fick’s first law of diffusion, and the diffusion coefficient for the type 1 collagen hydrogel was 3.8 × 10^−10^ m^2^ s^−1^. Results were obtained by establishing the concentration of the pro-inflammatory cytokines and the time-dependent range between the macrophage channel and the IVD cell channel. Automated microscopic time-lapse images of individual hAF cells grown for 72 h were acquired using a live cell imaging system (Nowon, Seoul, Korea). The cytoplasmic area and migration speed of cells in the acquired cell images was determined using ImageJ.

### Statistical analyses

The results are expressed as the relative protein expression ± standard error for the micro-EI stimulation experiments and kinetic analysis of six experiments with individual cells. The data were subjected to analysis of variance with One-way analysis of variance (ANOVA) and Bonferroni’s correction post hoc test were used to assess the differences in the experimental groups. Differences with P < 0.05 were assumed to be statistically significant. All statistical analyses were performed using SPSS software ver. 21.3 (SPSS, Inc., Chicago, IL, USA).

## Results

### Activated macrophage-like cells have a high capacity for secretion of pro-inflammatory cytokines in the micro-EI chip

**Figure 2:**
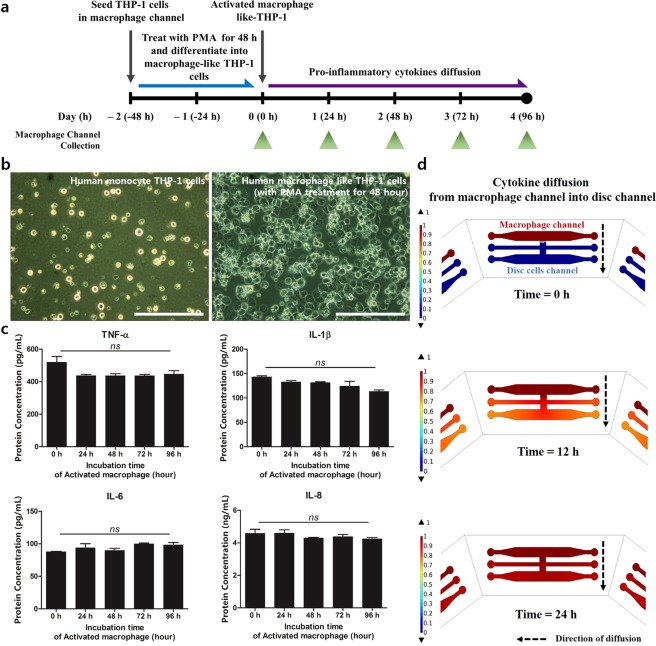
Differentiation of monocyte THP-1 cells and cytokines secretion by activated macrophage-like cells. (**a**) Pro-inflammatory cytokines collection process. (**b**) Representative images of monocyte THP-1 cells differentiated into activated macrophage-like THP-1 cells. Human monocyte THP-1 cells treated with PMA for differentiation for 48 h. **(c**) Production of inflammatory cytokines secreted by activated macrophage-like THP-1 cells for 96 h. (**d**) Characterization of the diffusion profile of applied macrophage derived-soluble factors over time. Values are mean ± SEM of five or six independent experiments. Blue corresponds to a normalized concentration of a soluble factor = 0, whereas red corresponds to 1.0. *ns = *no significant difference. The scale bar = 100 μm.

To assess the levels of the inflammatory activities of macrophages in the progression of IVD degeneration on the micro-EI-chip, we firstly cultured monocyte THP-1 cells with PMA in the macrophage channel and induced the differentiation into activated macrophage-like cells within 48 h. We then measured the amounts of the secreted proinflammatory mediators in conditioned medium from the IVD cells channel in the absence of hAF cells every 24 h for up to 96 h (Fig. 2a). Representative images showed that approximately 87% of monocyte THP-1 cells differentiated into activated macrophage cells by the 48-h PMA treatment (Fig. 2a: PMA treatment, and 2b). The levels of pro-inflammatory cytokines were stably maintained in the channel for 96 h. However, IL-1β tended to decrease slightly at 96 h time (Fig. 2c). Hence, the duration of the experiment was set at 72 h (Fig. 1d; whole experimental process). The activated macrophages secreted TNF-α, IL-1β, IL-6, and IL-8, which could induce inflammatory and degenerative conditions in hAF cells. After, we simulated the diffusion of these cytokines from the macrophage channel into the IVD cells channel. Cytokines from the activated macrophages transferred into the IVD cells channel by approximately 98% of the normalized concentration within 24 h (Fig. 2d and Supplementary Fig. S4). Subsequently, right after the macrophage-like cell activation at 48 h, we seeded hAF into the channel of the IVD cells to provide an inflammatory microenvironment for 24 h (Fig. 1d; whole experimental process).

To sum up, we demonstrated that THP-1 cells were transformed into activated macrophage-like cells and the levels of secretion of four major proinflammatory mediators remained stable for 72 h. The results also showed that the micro-EI-chip could transfer the produced cytokines to the IVD cells channel effectively within 24 h.

### Pro-inflammatory cytokines derived from activated macrophages upregulate inflammatory mediators and ECM-modifying enzymes in hAF cells

**Figure 3:**
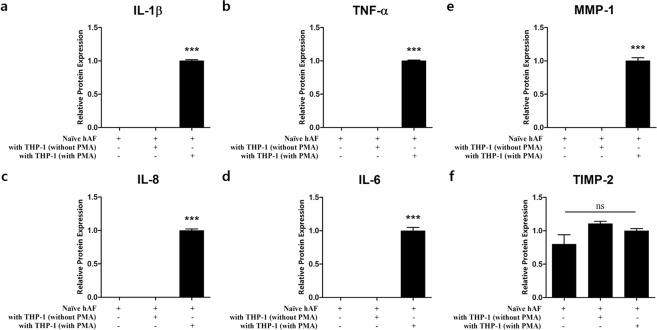
Production of inflammatory mediators and ECM enzymes on hAF co-cultured with THP-1 cells (with or without PMA). Production of **(a**) IL-1β, **(b)** TNF-α, **(c)** IL-8, and **(d)** IL-6 on hAFs induced by macrophage-like THP-1 cells. Production of ECM-modifying enzymes **(e)** MMP-1 and **(f)** TIMP-2. Values are Relative Expression (±SEM) of five or six independent experiments. Abbreviations are: ECM, extracellular matrix; hAFs, human annulus fibrosus cells; TNF-α, tumour necrosis factor-alpha, IL-1β, interleukin-1 beta; IL-6, interleukin 6; IL-8, interleukin 8; MMP-1, matrix metalloproteinase-1; and TIMP-2, tissue inhibitor of metalloproteinase-2.

After observing that inflammatory mediators and ECM-modifying enzymes in disc tissue are upregulated during intervertebral disc degeneration, we examined whether activated macrophages induce the production of these factors in hAFs by paracrine signaling (i.e., without direct contact). First, we detected the expression of inflammatory mediators in hAFs after cytokine induction by THP-1 cells with or without PMA for 24 h. THP-1 cells (without PMA) had no effect on the induction of inflammatory factors in hAF cells. However, the PMA treated THP-1 cells showed markedly increased concentrations of all inflammatory factors, including TNF-α, IL-1β, IL-6, and IL-8 in hAF cells (Fig. 3a–d). Consistent with this finding, we also detected the significantly increased production of MMP-1 in activated THP-1 co-cultured hAF cells, which is a crucial regulator of matrix degradation and cellular migration. However, TIMP-2, an endogenous inhibitor of MMP-1, was not significantly different between inflamed hAFs and naïve hAF (P > 0.05) (Fig. 3e and f). These results demonstrate that pro-inflammatory cytokines derived from macrophage-like cells regulate the balance between the catabolic and anabolic response of hAFs.

### Micro-EI stimulation inhibits expression of inflammatory mediators and NF-κB p50 protein activation in hAFs

To investigate the effects of micro-EI stimulation on inflammatory mediators, we treated hAFs using micro-EI at different frequencies (100, 200, and 300 Hz) at 150 mV/mm. These settings reflect those reported within the human body for biological activation.

**Figure 4:**
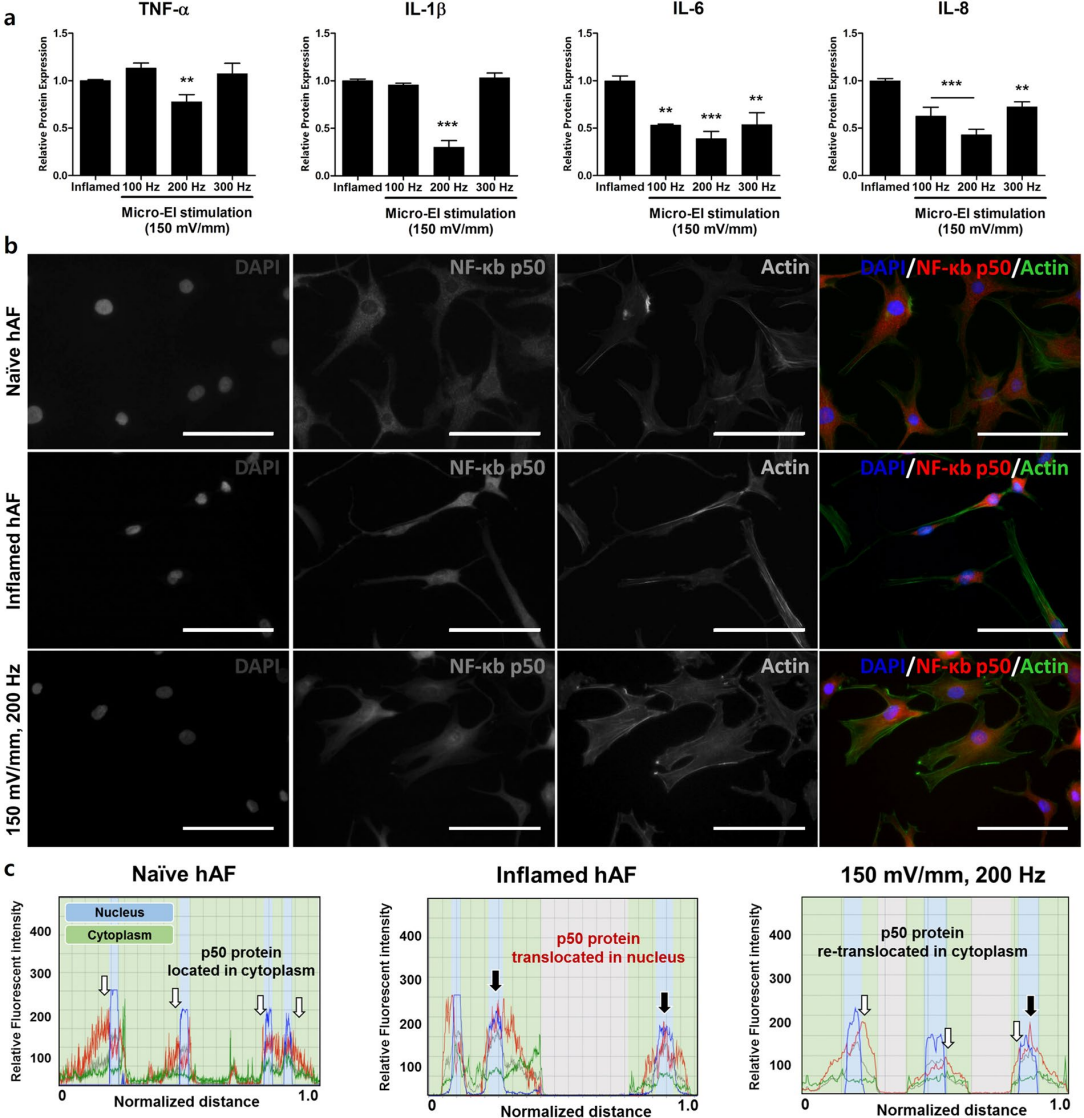
Production of inflammatory mediators on inflamed hAFs stimulated by micro-EI, mediated via the expression and translocation NF-κB p50. (**a**) Expression of inflammatory mediators on hAFs stimulated with micro-EI. (**b**) Fluorescence image of NF-κB p50 protein levels in hAF stimulated by micro-EI at 200 Hz with 150 mV/mm. (**c**) Quantification of the fluorescence intensity and preferential distribution of NF-κB p50 protein levels in hAFs. Values are Relative Expression (±SEM) of five or six independent experiments. Abbreviations are: TNF-α, tumour necrosis factor-alpha, IL-1β, interleukin-1 beta; IL-6, interleukin-6; IL-8, interleukin-8; micro-EI, micro-electrical impulse; hAFs, human annulus fibrosus cells; and NF-κB, nuclear factor-kappa B.

We used ELISA to measure TNF-α, IL-1β, IL-6, and IL-8, which are mediators involved in the inflammatory response. Micro-EI stimulated-hAFs displayed the dramatically attenuated secretion of these cytokines. All micro-EI frequencies showed inhibitory effects on IL-6 and IL-8 levels. Particularly, micro-EI stimulation with 150 mV/mm at 200 Hz resulted in strong attenuation of TNF-α, IL-1β, IL-8, and IL-6 (Fig. 4a). In addition, micro-EI stimulation at 150 mV/mm 200 Hz did not affect the inflammatory cytokines expression of naïve hAF cells, but 200 Hz showed a strong anti-inflammatory effect on the inflamed hAF cells compared with 100 Hz and 300 Hz (supplementary Fig. S5). The inflammatory attenuation effect on TNF-α, IL-1β, IL-6, and IL-8 from micro-EI stimulation persisted for 48 h after termination of stimulation (Supplementary Fig. S6). After observing that activated macrophages secreted a variety of mediators and enzymes affecting hAFs, we next investigated the intrinsic mechanism by which the soluble factors derived from activated macrophages regulate the production of inflammatory mediators and ECM enzymes. We examined the induction of NF-κB p50 activation by activated macrophages in hAF cells, in which NF-κB exerts its transcriptional activity by translocation into the nucleus to promote inflammatory and catabolic responses. We predicted that the downregulation of inflammatory cytokines would be accompanied by decreased NF-κB p50 protein activation. The immunofluorescence images revealed that the preferential distribution of the p50 protein in the nucleus rather than in the cytoplasm in inflamed hAFs. However, with micro-EI stimulation at 150 mV/mm with 200 Hz, most p50 re-translocated to the cytoplasm (Fig. 4b). Quantitatively, p50 activity calculated from the average intensity value in inflamed hAFs was markedly reduced by micro-EI stimulation at 150 mV/mm with 200 Hz. Activity was the highest in the cytoplasm (Fig. 4c). Collectively, these data demonstrate that micro-EI stimulation inhibits the production of inflammatory mediators and decreases the activation of NF-κB p50.

### Micro-EI stimulation inhibits the expression of ECM enzymes and modulates the kinetic characteristics in degenerated hAFs

**Figure 5:**
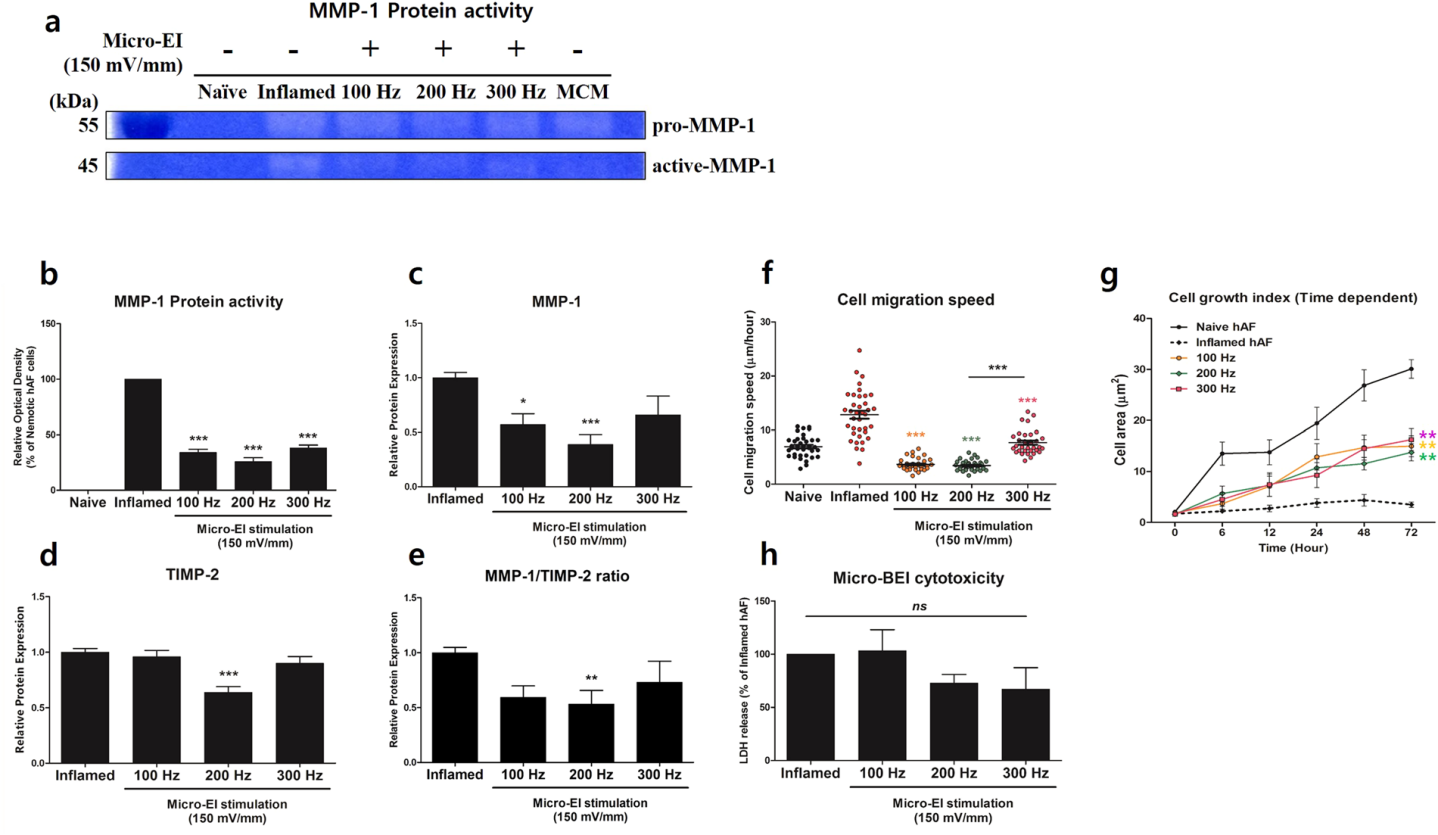
Production of ECM-modifying enzymes and kinetic characteristics of inflamed hAFs stimulated by micro-EI. (**a**) MMP-1 activity analysed by collagen zymography. **(b)** Relative optical density of MMP-1 activity from zymography. **(c**, **d)** Production of MMP-1 **(c)** and TIMP-2 **(d)**. **(e)** Production ratio of MMP-1/TIMP-2. **(f)** Rate of cell migration. **(g)** Rate of cell growth. **(h)** LDH cytotoxicity of micro-EI stimulation on hAF. Values are Relative Expression (±SEM) of five or six independent experiments. Abbreviations are: ECM, extracellular matrix; micro-EI, electrical impulse; hAFs, human annulus fibrosus cells; MMP-1, matrix metalloproteinase-1; TIMP-2, tissue inhibitor of metalloproteinase-2; and LDH, lactate dehydrogenase.

In healthy discs, the rates of ECM synthesis and breakdown are in equilibrium. MMPs expressed at a low level in non-degenerate discs are involved in normal tissue repair and remodelling. However, during degeneration, the expressions of these ECM-modifying enzymes are upregulated in hAFs. Changes in the expression of ECM-modifying enzymes affect morphological features and migration speed of cells due to cell-ECM interactions. Thus, we next evaluated the expression pattern of ECM-modifying enzymes MMP-1 and TIMP-2 in hAFs stimulated with micro-EI and cell kinetic characteristics of degenerated hAFs using a live cell imaging system. Interestingly, we found that micro-EI at 200 Hz with 150 mV/mm significantly inhibited MMP-1 and TIMP-2 expression, while the other micro-EI groups were not affected (Fig. 5c and d). Additionally, to examine the biological activity of MMP-1, we measured its ability to cleave interstitial fibrillary collagen by zymography. All frequencies of micro-EI attenuated the transformation of MMP-1 from the pro-form to the active-form (Fig. 5a and b and Supplementary Fig. S7). Micro-EI stimulation also significantly downregulated the MMP-1/TIMP-2 ratio (Fig. 5e). These effects of micro-EI were maintained for 48 h after termination of stimulation (Supplementary Fig. S8).

Collectively, these results demonstrate that micro-EI stimulation inhibited ECM degradation by downregulating the protein level and activity of MMP-1. Furthermore, micro-EI stimulation shifted the ratio of MMP-1/TIMP-2 in hAF cells from a catabolic to an anabolic response, which is responsible for the balance between ECM synthesis and breakdown in IVD.

We also hypothesized that expression changes of these enzymes would facilitate the loosening of cell-matrix and cell-cell interactions within the degenerative disc, resulting in the migration of hAFs into the defect. Our kinetic data showed that the rate of individual cell migration was significantly increased in inflamed hAFs compared to that of naïve hAFs (Fig. 5f). Furthermore, inflamed hAFs exhibited a decreased cell cytoplasm area and low growth rate (Fig. 5g). Interestingly, similar to the effect on ECM protein levels, all frequencies of micro-EI stimulation showed reparative effects on the kinetic characteristics of inflamed hAFs. Additionally, micro-EI stimulation showed no significant toxicity (Fig. 5h).

Collectively, these data suggested that micro-EI stimulation has positive effects on degenerative hAFs induced by pro-inflammatory cytokines derived from macrophages. Additionally, the frequency of micro-EI affects the differential expression of ECM enzymes and inflammatory mediators in hAFs.

## Discussion

Microengineered biomimetic organ-on-chip technology has advantages over animal models because it enables direct real-time visualisation because of its optical transparency; it can recapitulate complex interactions between different types of cells *in vivo* using compartmentalised channel designs for co-culture; and it can control soluble and insoluble factors, such as cytokines, chemokines, growth factors, ECM, and intercellular junctions simultaneously^33^.

In our previous study, we showed the effects of ES on hAF cells treated only with recombinant IL-1β. However, the present study focused on mimicking the actual inflammatory environment by co-culture of human monocytes and hAF cells followed by various analyses of the anti-inflammatory effect of ES. We developed a 3D printed micro-EI stimulation chip to apply electrical stimulation in IVD. The micro-EI-chip platform has several novel features: 1) ability for various molecular biological analyses because of the multi-chambers in a single chipset; 2) live and transparently observable multi-coculture of different types of cells; 3) the capacitive polydimethylsiloxane (PDMS)-coated electrode allows not only precise ES through adjustable micro-EI strength and frequency, but also does not delaminate the metal layer on the electrodes; and 4) because of the use of 3D printing technology, the results are reproducible under similar conditions, and the system is fast and cost-effective. Using this chipset, we showed that: 1) inflammatory mediators and matrix-modifying enzymes, such as TNF-α, IL-1β, IL-6, IL-8, MMP-1, and TIMP-2, were upregulated in macrophage mediated-inflamed human AF cells^34^; 2) the kinetic characteristics of hAFs were also modulated under degenerative conditions; 3) these phenotypic and genotypic changes were mediated by NF-κB p50 translocation and activation; and 4) micro-EI stimulation inhibited these inflammatory molecules and catabolic enzymes in inflamed AF cells induced by macrophages, particularly at 200 Hz with 150 mV/mm.

Macrophages play central roles in the inflammatory response in the IVD^35,36^. Additionally, the microvascular system, which acts as a track for the infiltration and activation of macrophages, invades the inner disc tissue during disc degeneration^37^. Increasing evidence has indicated that several inflammatory cells, such as macrophages or lymphocytes, are infiltrated in herniated discs. Therefore, we hypothesised that soluble pro-inflammatory cytokines secreted by activated macrophages would play important roles in the progression of disc degeneration. Indeed, our study showed that activated macrophages secrete pro-inflammatory cytokines, including TNF-α and IL-1β, which are activators of the NF-κB and mitogen-activated protein kinase signalling pathways in various tissues^38^. Furthermore, in the co-culture study, we showed that these cytokines induce the secretion of inflammatory mediators and catabolic enzymes, such as IL-6, IL-8, MMP-1, and TIMP-2, in human AF cells. Moreover, these expression changes were positively correlated with the activation and translocation of NF-κB p50.

NF-κB is a nuclear factor that possesses transcriptional regulatory activity and is present in the cells of various tissues. Normally, NF-κB, which includes a p65-p50 heterodimer, is sequestered in the cytoplasm because it binds to inhibitor of nuclear factor κB^38^. However, pro-inflammatory cytokines, including TNF-α and IL-1β, cause the ubiquitylation and phosphorylation of inhibitor of nuclear factor κB and its degradation, allowing the NF-κB heterodimer to translocate into the nucleus where it promotes the expression of several inflammatory catabolic genes. In the final stage, the inducible inflammatory cytokines play a major role in the sensitisation of nerve endings and irritation of dorsal root ganglion-derived nociceptive channel activity, including ASIC-3 and TrpV1^2^. In the regulation of catabolic enzymes, increased expression of IL-1β and TNF-α induce upregulation of genes encoding ECM-degrading enzymes, including MMPs and TIMPs^37,39^. Furthermore, under degenerative conditions, these enzymes induce proteolytic cleavage of aggrecan and alter various kinetic characteristics, such as migration, growth, and structural modification, resulting in histological inhomogeneity and enhancing nerve invasion and nociceptive responses in disc cells^40^. During this process, the IVD tissues generate electric signals for biochemical signal transduction to nerve fibres and are controlled by endogenous/exogenous electric signals. Various ES types can affect cell proliferation and differentiation by influencing the expression of relevant genes and proteins. Therefore, we hypothesised that extrinsic ES modulates protein levels in human AF cells. Indeed, we found that ES controls the expression of biochemical signal cascades. Additionally, transcutaneous electrical nerve stimulation (TENS) and functional ES have been increasingly studied as alternative therapies. Some studies have shown that ES reduces pain intensity compared to in control groups in a variety of disorders, indicating effective analgesia^41–44^. One study demonstrated that the application of TENS on incised wounds improves healing by increasing growth factors (epidermal growth factor, platelet-derived growth factor-A, and fibroblast growth factor-2) in the dermis and epidermis. Additionally, TENS application resulted in decreased TNF-α, IL-1β, and IL-6 immunoreactions in incised wounds^42^. Another study reported that TENS treatment attenuates hyperalgesia and inhibits spinal extracellular signal-regulated kinase 1/2-cyclooxygenase-2 pathway activation in rats^45^. A clinical study showed that functional ES improves endothelial function and has anti-inflammatory effects by reducing TNF-α, IL-10, soluble intercellular adhesion molecule, and soluble vascular cell adhesion molecule^43^. These data are consistent with our findings showing decreased expression of inflammatory cytokines in AF cells after ES application. Furthermore, previous studies demonstrated that several parameters, including the dose and frequency of ES, determines whether the cells respond^18^. Overall, these ES-related studies suggest that the molecular regulatory effects of ES can be applied to IVD degeneration associated with chronic pain.

Our results showed that micro-EI stimulation with 150 mV/mm 200 Hz for 48 h dramatically decreased inflammatory mediators, such as TNF-α, IL-1β, IL-6, and IL-8, and ECM-modifying enzymes, including MMP-1, TIMP-2, and the MMP-1/TIMP-2 ratio. Additionally, downregulation of TNF-α, IL-1β, IL-6, IL-8, and MMP-1 and the MMP-1/TIMP-2 ratio were maintained for 48 h after the termination of stimulation. However, IL-1β and TIMP-2 factors increased at 48 h following termination of micro-EI stimulation (Supplementary Fig. S8). In this study, there were significant effects of micro-EI on the production of TNF-α and IL-1β. Other research groups reported that upregulation of the IL-1 receptor type 1 (IL1R1) gene by 80% in degenerative or herniated discs compared to in non-degenerative discs by immunohistochemical analysis. In contrast, the gene encoding TNF receptor type 1 reportedly did not differ among degenerative and non-degenerative discs^2,46^. Correspondingly, IL-1β may be a more important key inflammatory factor mediating modulation by micro-EI stimulation in IVD degeneration. The capability of ES to ameliorate degenerated discs and the frequency ranges at which it is effective are unknown. We found that micro-EI stimulation abolishes macrophage-induced translocation and activation of NF-κB p50 protein at 200 Hz with 150 mV/mm, which may represent the most effective profile for inhibiting inflammatory and catabolic enzymes.

Recently, the pharmaceutical companies GlaxoSmithKline and Google invested €630 million to create new bioelectronic medicines as emerging alternative therapeutic technologies, named as electroceuticals, which are based on modulations of electrical signalling patterns of the peripheral nervous system^47–50^. The US National Institutes of Health announced a US$ 248 million effort to map the electrical wiring of the human body and thereby develop such devices^51,52^. Hence, our micro-EI-chip platform can be utilized for basic research of electroceuticals.

## Conclusions

In the present study, a 3D printed micro-EI stimulation chip was developed as a cost-effective *in vitro* model for hit-to-lead and lead optimization that can more reliably predict novel therapeutic targets for treating symptomatic IVD disease. Using the micro-EI-chip, we applied micro-EI to degenerative inflamed hAF cells to mimic the microenvironment during symptomatic disc degeneration and performed molecular analyses to assess anti-inflammatory effects and cell kinetics, such as those changes in the cell cytoplasm area and cell migration, respectively. The results indicate that the micro-EI modulates pro-inflammatory cytokines and ECM-modifying enzymes. These features will promote the clinical application of EI stimulation in disc disease.

## Supplementary information

Supplementary information for “Electrical impulse effects on degenerative human annulus fibrosus model to reduce disc pain using micro-electrical impulse-on-a-chip”

## References

[1] Gellhorn AC, Katz JN, Suri P (2013). Osteoarthritis of the spine: the facet joints. Nat Rev Rheumatol..

[2] Risbud MV, Shapiro IM (2014). Role of cytokines in intervertebral disc degeneration: pain and disc content. Nat Rev Rheumatol..

[3] Molinos M (2015). Inflammation in intervertebral disc degeneration and regeneration. J R Soc Interface..

[4] Belavy DL (2017). Running exercise strengthens the intervertebral disc. Sci Rep..

[5] Nguyen QT, Jacobsen TD, Chahine NO (2017). Effects of inflammation on multiscale biomechanical properties of cartilaginous cells and tissues. ACS Biomater. Sci. Eng..

[6] Chan SC, Ferguson SJ, Gantenbein-Ritter B (2011). The effects of dynamic loading on the intervertebral disc. Eur. Spine J..

[7] Chooi WH, Chan BP (2016). Compression loading-induced stress responses in intervertebral disc cells encapsulated in 3D collagen constructs. Sci. Rep..

[8] Yamauchi K (2009). Nerve growth factor of cultured medium extracted from human degenerative nucleus pulposus promotes sensory nerve growth and induces substance p *in vitro*. Spine (Phila. Pa. 1976).

[9] Abe Y (2007). Proinflammatory cytokines stimulate the expression of nerve growth factor by human intervertebral disc cells. Spine (Phila. Pa. 1976).

[10] Richardson SM (2012). Degenerate human nucleus pulposus cells promote neurite outgrowth in neural cells. PLoS One.

[11] Yan J (2016). Inhibition of cystathionine beta-synthetase suppresses sodium channel activities of dorsal root ganglion neurons of rats with lumbar disc herniation. Sci. Rep..

[12] Huang BR (2017). EGFR is a pivotal regulator of thrombin-mediated inflammation in primary human nucleus pulposus culture. Sci. Rep..

[13] Moon HJ (2012). Annulus fibrosus cells interact with neuron-like cells to modulate production of growth factors and cytokines in symptomatic disc degeneration. Spine (Phila. Pa. 1976).

[14] Kazezian Z (2015). Gene expression profiling identifies interferon signalling molecules and IGFBP3 in human degenerative annulus fibrosus. Sci. Rep..

[15] Wiet MG (2017). Mast cell-intervertebral disc cell interactions regulate inflammation, catabolism and angiogenesis in discogenic back pain. Sci. Rep..

[16] Matta A, Karim MZ, Isenman DE, Erwin WM (2017). Molecular therapy for degenerative disc disease: clues from secretome analysis of the notochordal cell-rich nucleus pulposus. Sci. Rep..

[17] Hu B (2016). Heme oxygenase-1 attenuates IL-1beta induced alteration of anabolic and catabolic activities in intervertebral disc degeneration. Sci. Rep..

[18] Kim JH (2013). Effect of biphasic electrical current stimulation on IL-1beta-stimulated annulus fibrosus cells using *in vitro* microcurrent generating chamber system. Spine (Phila. Pa. 1976).

[19] Zhao M (2006). Electrical signals control wound healing through phosphatidylinositol-3-OH kinase-gamma and PTEN. Nature.

[20] Pavesi A (2016). Engineering a 3D microfluidic culture platform for tumor-treating field application. Sci. Rep..

[21] Kim IS (2009). Novel effect of biphasic electric current on *in vitro* osteogenesis and cytokine production in human mesenchymal stromal cells. Tissue Eng. Part A.

[22] Miller SL, Coughlin DG, Waldorff EI, Ryaby JT, Lotz JC (2016). Pulsed electromagnetic field (PEMF) treatment reduces expression of genes associated with disc degeneration in human intervertebral disc cells. Spine J..

[23] Wang Z, Hutton WC, Yoon ST (2017). The effect of capacitively coupled (CC) electrical stimulation on human disc nucleus pulposus cells and the relationship between CC and BMP-7. Eur. Spine J..

[24] Park HJ, Rouabhia M, Lavertu D, Zhang Z (2015). Electrical stimulation modulates the expression of multiple wound healing genes in primary human dermal fibroblasts. Tissue Eng. Part A.

[25] Prasad A (2017). Static magnetic field stimulation enhances oligodendrocyte differentiation and secretion of neurotrophic factors. Sci. Rep..

[26] Funk RH, Monsees T, Ozkucur N (2009). Electromagnetic effects - From cell biology to medicine. Prog. Histochem. Cytochem..

[27] Rosenspire AJ, Kindzelskii AL, Simon BJ, Petty HR (2005). Real-time control of neutrophil metabolism by very weak ultra-low frequency pulsed magnetic fields. Biophys. J..

[28] Chen CF (2017). Higher-order power harmonics of pulsed electrical stimulation modulates corticospinal contribution of peripheral nerve stimulation. Sci. Rep..

[29] McCaughey EJ, Borotkanics RJ, Gollee H, Folz RJ, McLachlan AJ (2016). Abdominal functional electrical stimulation to improve respiratory function after spinal cord injury: a systematic review and meta-analysis. Spinal Cord.

[30] Kim JH, Studer RK, Sowa GA, Vo NV, Kang JD (2008). Activated macrophage-like THP-1 cells modulate anulus fibrosus cell production of inflammatory mediators in response to cytokines. Spine (Phila. Pa. 1976).

[31] Satsu H (2006). Induction by activated macrophage-like THP-1 cells of apoptotic and necrotic cell death in intestinal epithelial Caco-2 monolayers via tumor necrosis factor-alpha. Exp. Cell. Res..

[32] Kim JH, Studer RK, Vo NV, Sowa GA, Kang JD (2009). p38 MAPK inhibition selectively mitigates inflammatory mediators and VEGF production in AF cells co-cultured with activated macrophage-like THP-1 cells. Osteoarthritis Cartilage.

[33] Marx V (2015). Tissue engineering: organs from the lab. Nature.

[34] Johnson, Z. I., Schoepflin, Z. R., Choi, H., Shapiro, I. M. & Risbud, M. V. Disc in flames: Roles of TNF-alpha and IL-1beta in intervertebral disc degeneration. *Eur. Cell. Mater*. **30**, 104–116, discussion 116-107 (2015). 10.22203/ecm.v030a08 PMC4751407 26388614

[35] Wang J (2013). Tumor necrosis factor alpha- and interleukin-1beta-dependent induction of CCL3 expression by nucleus pulposus cells promotes macrophage migration through CCR1. Arthritis Rheum..

[36] Yang C (2016). Differential expression of p38 MAPK alpha, beta, gamma, delta isoforms in nucleus pulposus modulates macrophage polarization in intervertebral disc degeneration. Sci. Rep..

[37] Phillips KL (2015). Potential roles of cytokines and chemokines in human intervertebral disc degeneration: interleukin-1 is a master regulator of catabolic processes. Osteoarthritis Cartilage.

[38] Catrysse L, van Loo G (2017). Inflammation and the metabolic syndrome: the tissue-specific functions of NF-kappaB. Trends Cell Biol..

[39] Vo NV (2013). Expression and regulation of metalloproteinases and their inhibitors in intervertebral disc aging and degeneration. Spine J..

[40] Binch AL (2014). Expression and regulation of neurotrophic and angiogenic factors during human intervertebral disc degeneration. Arthritis Res. Ther..

[41] do Carmo Almeida TC (2017). Effects of transcutaneous electrical nerve stimulation (TENS) on proinflammatory cytokines: protocol for systematic review. Syst. Rev..

[42] Gürgen SG, Sayın O, Çetin F, Yücel AT (2014). Transcutaneous electrical nerve stimulation (TENS) accelerates cutaneous wound healing and inhibits pro-inflammatory cytokines. Inflammation.

[43] Karavidas AI (2006). Functional electrical stimulation improves endothelial function and reduces peripheral immune responses in patients with chronic heart failure. Eur. J. Cardiovasc. Preve. Rehabil..

[44] Ratajczak B, Hawrylak A, Demidaś A, Kuciel-Lewandowska J, Boerner E (2011). Effectiveness of diadynamic currents and transcutaneous electrical nerve stimulation in disc disease lumbar part of spine. J. Back Musculoskelet. Rehabil..

[45] Fang J-F, Liang Y, Du J-Y, Fang J-Q (2013). Transcutaneous electrical nerve stimulation attenuates CFA-induced hyperalgesia and inhibits spinal ERK1/2-COX-2 pathway activation in rats. BMC Complement. Altern. Med..

[46] Le Maitre CL, Hoyland JA, Freemont AJ (2007). Catabolic cytokine expression in degenerate and herniated human intervertebral discs: IL-1beta and TNFalpha expression profile. Arthritis Res. Ther..

[47] Famm K, Litt B, Tracey KJ, Boyden ES, Slaoui M (2013). Drug discovery: a jump-start for electroceuticals. Nature.

[48] Koopman FA (2016). Vagus nerve stimulation inhibits cytokine production and attenuates disease severity in rheumatoid arthritis. Proc. Natl. Acad. Sci. USA.

[49] Onuora S (2016). Rheumatoid arthritis: Vagus nerve stimulation reduces RA severity in patients. Nat. Rev. Rheumatol..

[50] Birmingham K (2014). Bioelectronic medicines: a research roadmap. Nat. Rev. Drug Discov..

[51] Sinha G (2013). Charged by GSK investent, battery of electroceuticals advance. Nat. Med..

[52] Reardon S (2014). Electroceuticals spark interest: industry and academia invest in treating diseases by delivering electrical charges to nerves. Nature.

