# Supplementary material for: Electrical impulse effects on degenerative human annulus fibrosus model to reduce disc pain using micro-electrical impulse-on-a-chip
Source: Sci Rep. 2019 Apr 9;9:5827. doi: 10.1038/s41598-019-42320-9 (PMC6456732; doi:10.1038/s41598-019-42320-9)
Supplement: Supplementary file 1 — Supplementary information for “Electrical impulse effects on degenerative human annulus fibrosus model to reduce disc pain using micro-electrical impulse-on-a-chip” [file 41598_2019_42320_MOESM1_ESM.pdf]

Supplementary information for "Electrical impulse effects on degenerative human annulus fibrosus model to reduce disc pain using micro-electrical impulse-on-a-chip"

JaeHee Shin[1,+], MinHo Hwang[1,+], SeungMin Back[1], HyoGeun Nam[1], ChangMin Yoo[1], JeongHun Park[1], HyeongGuk Son[1], JaeWon Lee[1], HyunJung Lim[1], KwangHo Lee[2], HongJoo Moon[3], Joo Han Kim[3], HanSang Cho[4], and Hyuk Choi[1,*]

[1]Department of Medical Sciences, Graduate School of Medicine, Korea University, Seoul, Korea

[2]Department of Advanced Material Science and Engineering, College of Engineering, Kangwon National University, Chuncheon 25561, Korea

[3]Department of Neurosurgery, Guro Hospital, College of Medicine, Korea University, Seoul, Korea

[4]Department of Mechanical Engineering and Engineering Science, Department of Biological Sciences, Center for Biomedical Engineering and Science, Nanoscale Science Program, University of North Carolina at Charlotte, 28223, USA

Figure S1

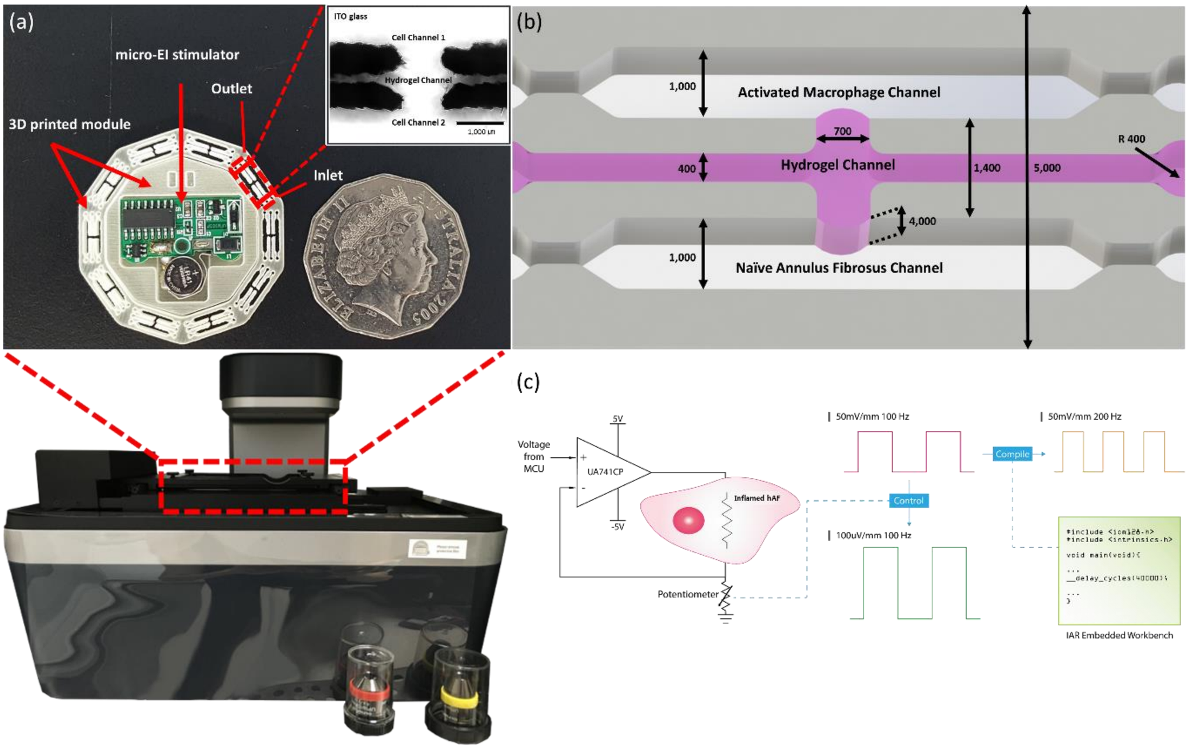

Figure S1. Micro-EI-chip platform. (a) Micro-EI-chip platform designed for culturing and stimulating cells and live cell imaging. **(b)** Schematic diagram of channel. **(C)** Micro-EI strength and frequency control profile.

Figure S2

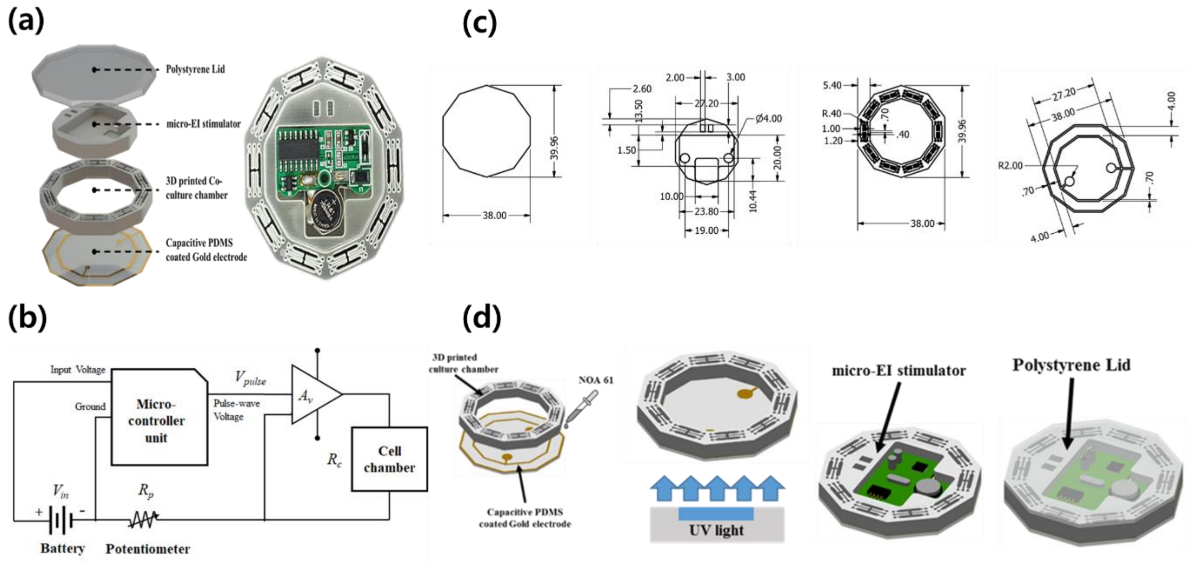

Figure S2. 3D printed parts and Assembly of the device. (a) 3D view of micro-EI chip. **(b)** micro-EI strength and frequency control profile. **(c)** Detail of 3D parts design map. **(d)** Assembly of the device. (1) Binding of the gold electrode ITO glass and 3D printed cell chamber is done using NOA 61 (Norland products, Inc., USA) and by curing for 30 min with UV illumination at a wavelength of 365 nm. (2) The micro-EI stimulator and 3D printed cell chamber are assembled. (3) The polystyrene lid is attached to the device. The device is ready for use in experiments.

Figure S3

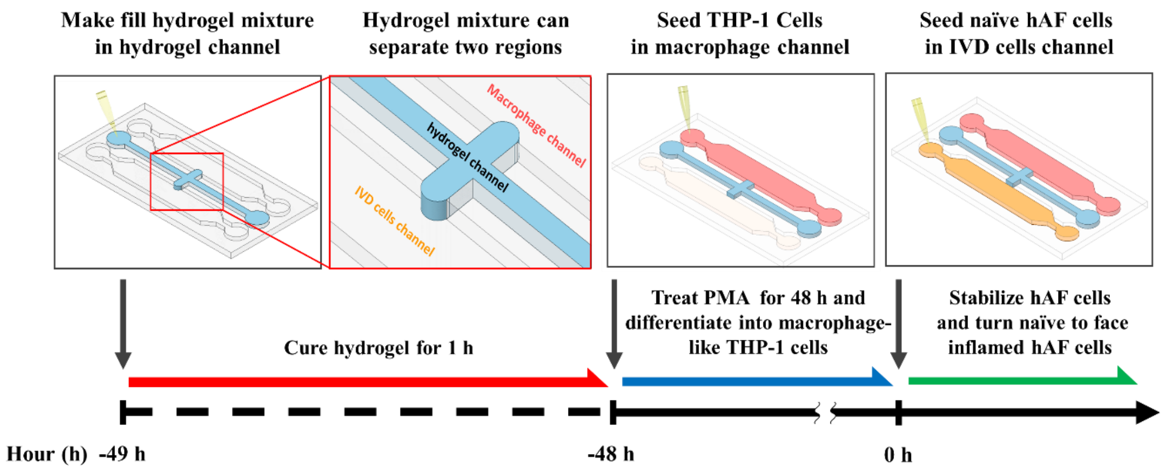

Figure S3. Hydrogel filling process. The hydrogel mixture (type 1 collagen, 2.0 mg/mL) is used to fill the hydrogel channel on Day -2 (-48 h) as an intervention that allows the diffusion of pro-inflammatory cytokines from macrophages to the channel of the IVD cell. The hydrogel mixture also separates the region between the macrophages and the channel. After 1 h, the hydrogel mixture is cured, and phorbol myristate acetate (PMA) treated THP-1 cells are seeded in the macrophage channel to allow the differentiation of THP-1 cells to macrophage-like THP-1 cells from Day -2 (-48 h) to Day 0 (0 h).

Figure S4

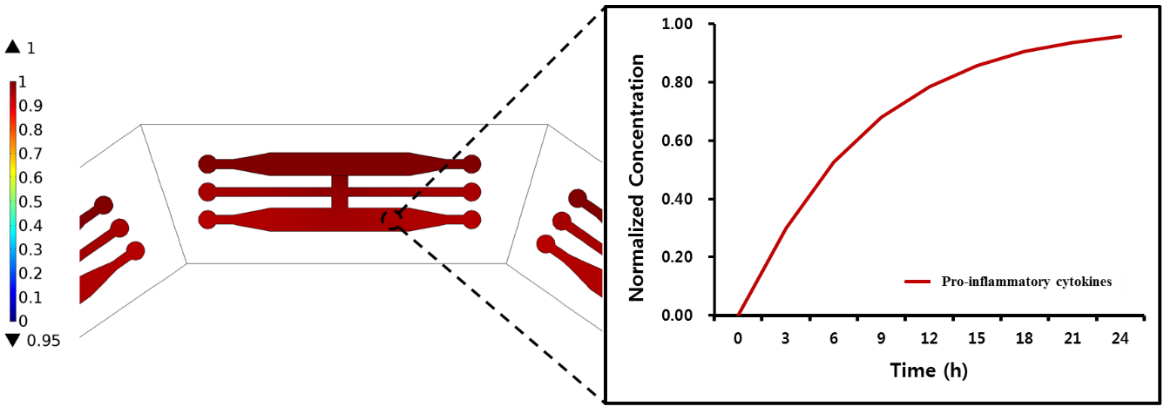

Fick's first law of diffusion	Variable description	Symbol	Range
$J_0(t) = -D\left(\frac{dC_i}{dx}(0,t)\right)$	Time	T	0 to 24 h
	Diffusion coefficient in hydrogel channel	D	3.8×10^{-10} m^2 s^{-1}
	Pro-inflammatory cytokines Concentration	C_i	0 to 1 mol m^{-3}
	Hydrogel channel length	x	1400 μm
	Diffusion flux	J	0 to 1 mol m^{-3} h^{-1}

Figure S4. Rate of concentration change of pro-inflammatory cytokines in the IVD cell channel. Pro-inflammatory cytokines diffuse into the IVD channel, which is the 24-h normalized concentration of pro-inflammatory cytokines.

Figure S5

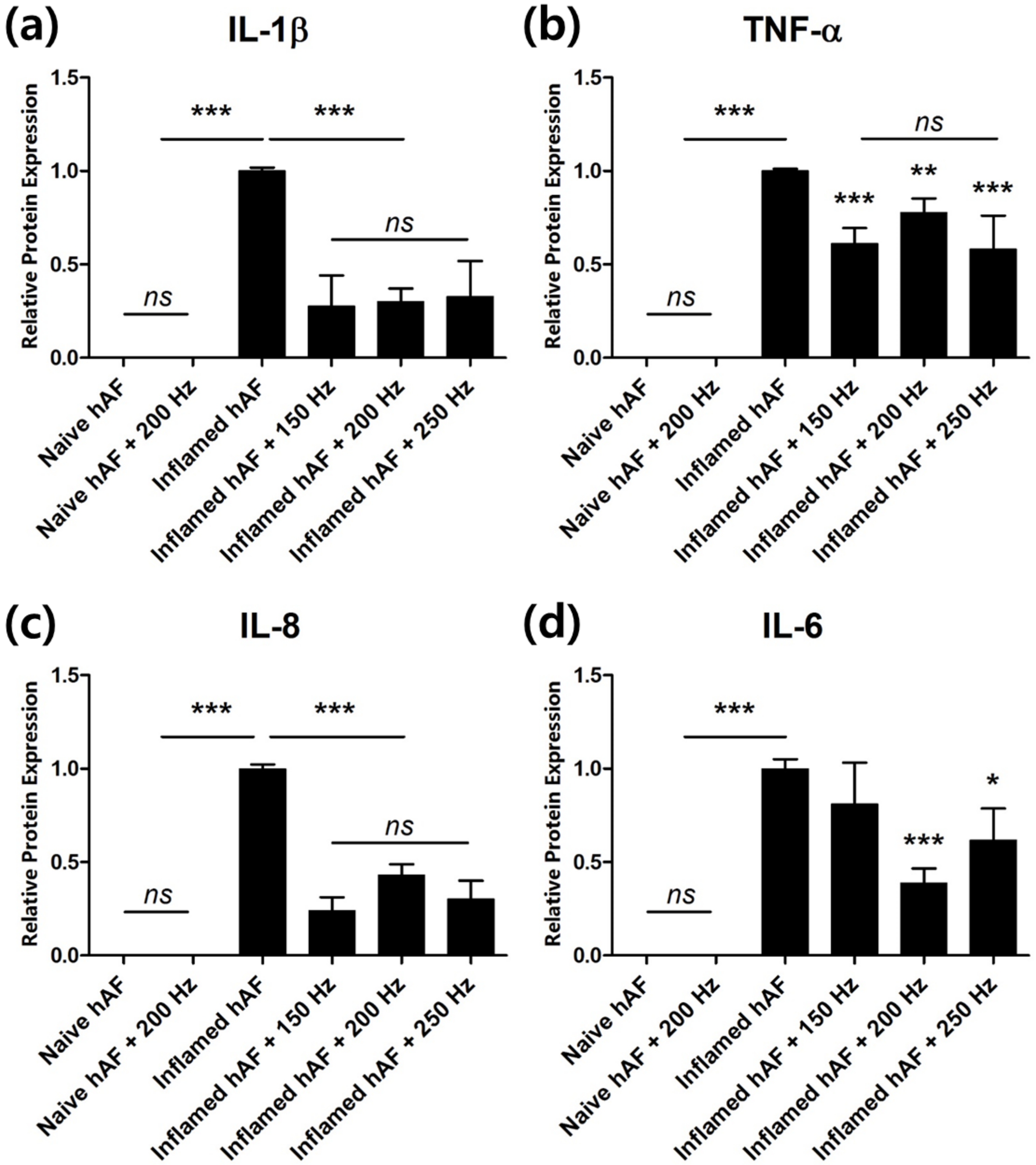

Figure S5. Concentration change of pro-inflammatory cytokines between 100 and 300 Hz of micro-EI stimulation of hAF cells. (a) IL-1β, **(b)** TNF-α, **(c)** IL-8, and **(d)** IL-6. Values are Relative Expression (± SEM) of three or four independent experiments. *P < 0.05 versus inflamed hAF cells, **P < 0.01 versus inflamed hAF cells, ***P < 0.001 versus inflamed hAF cells, and the line indicates comparison with each group.

Figure S6

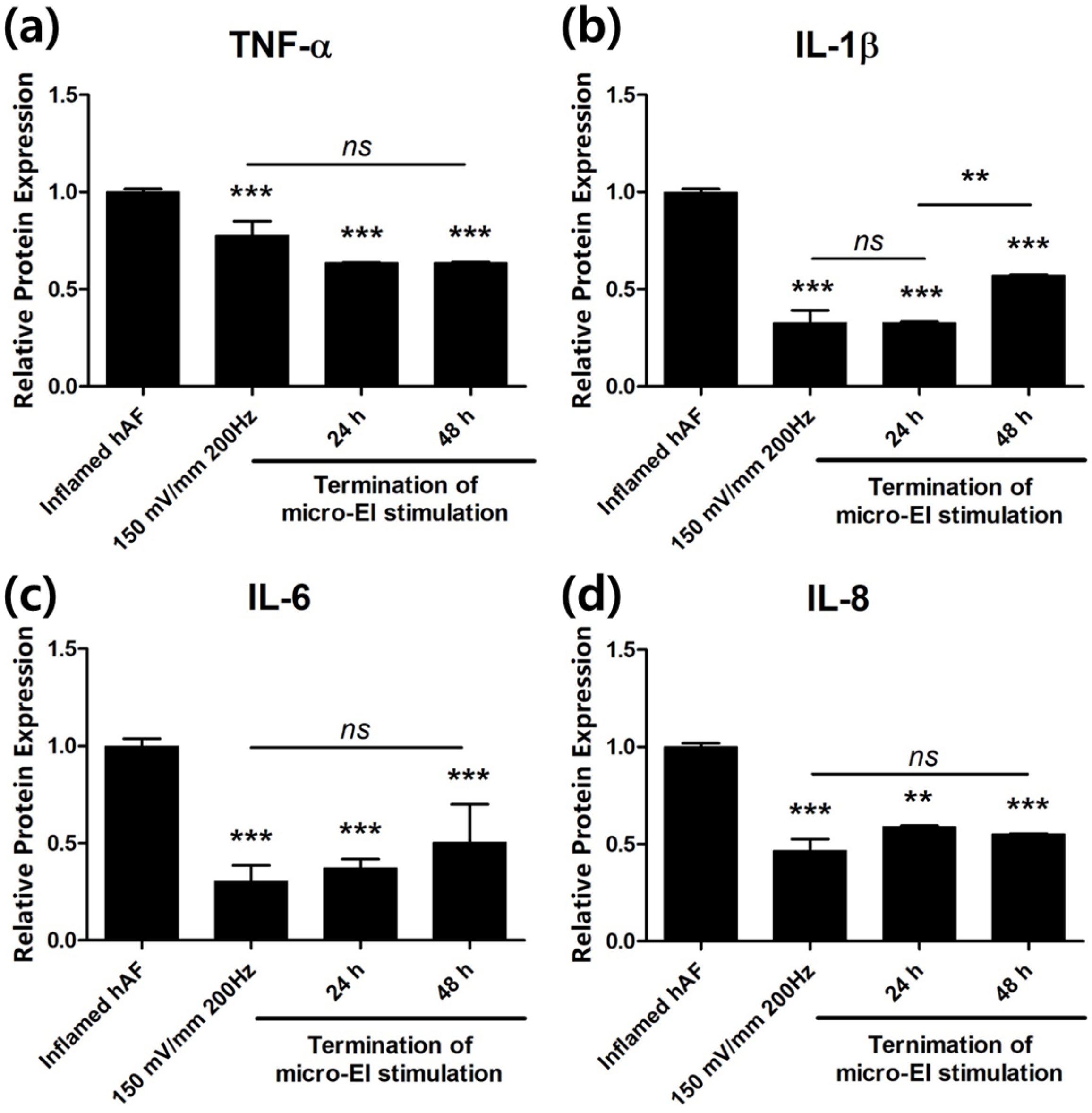

Figure S6. Production of inflammatory mediators after termination of micro-EI stimulation for 48 h. Expression of inflammatory mediators on hAFs. (a) TNF-α, **(b)** IL-1β, **(c)** IL-6, and **(d)** IL-8. Values are Relative Expression (± SEM) of three or four independent experiments. $^{**}P < 0.01$ versus inflamed hAF cells, $^{***}P < 0.001$ versus inflamed hAF cells, and the line indicates comparison with each group.

Figure S7

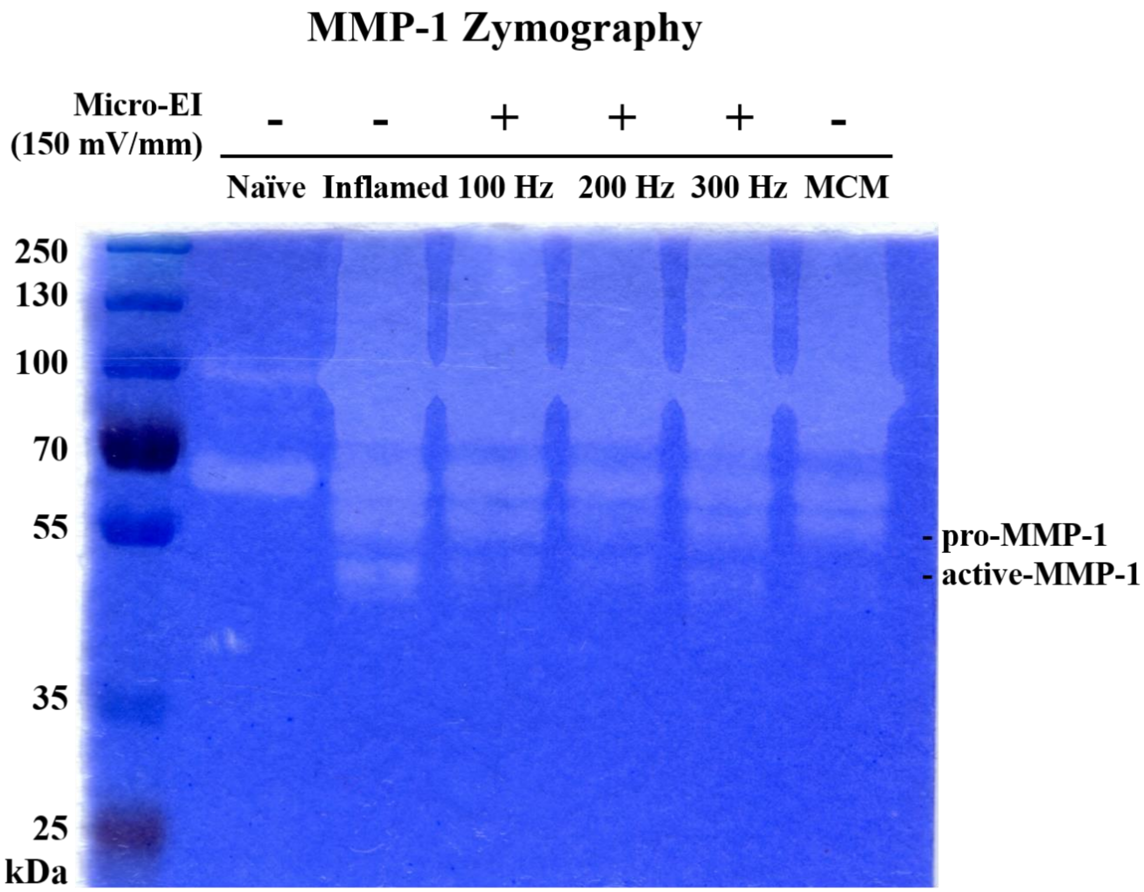

Figure S7. Full-length MMP-1 zymography.

Figure S8

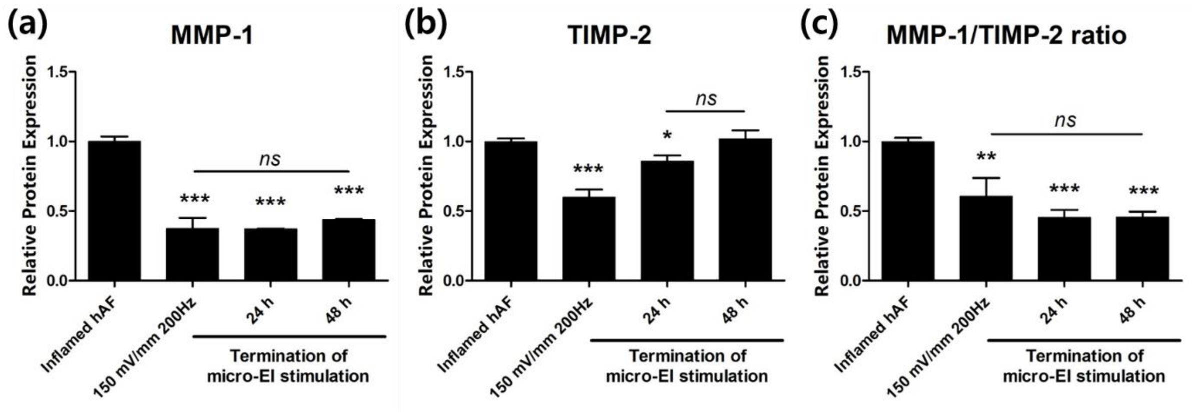

Figure S8. Production of ECM-modifying enzymes after termination of micro-EI stimulation for 48 h. (a) Production of MMP-1 and **(b)** TIMP-2. **(c)** Production ratio of MMP-1/TIMP-2 ratio. Values are Relative Expression (± SEM) of three or four independent experiments. *P < 0.05 versus inflamed hAF cells, **P < 0.01 versus inflamed hAF cells, ***P < 0.001 versus inflamed hAF cells, and the line indicates comparison with each group.
